# Publisher Correction: Smac mimetics synergize with immune checkpoint inhibitors to promote tumour immunity against glioblastoma

**DOI:** 10.1038/ncomms16231

**Published:** 2018-07-18

**Authors:** Shawn T. Beug, Caroline E. Beauregard, Cristin Healy, Tarun Sanda, Martine St-Jean, Janelle Chabot, Danielle E. Walker, Aditya Mohan, Nathalie Earl, Xueqing Lun, Donna L. Senger, Stephen M. Robbins, Peter Staeheli, Peter A. Forsyth, Tommy Alain, Eric C. LaCasse, Robert G. Korneluk

Nature Communications 8: Article number: 14278; DOI: 10.1038/ncomms14278 (2017); Published 02 15 2017, Updated 07 18 2018

The original HTML version of this Article omitted the article number; it should have been ‘14278’. This has now been corrected in the HTML version of the Article. The PDF version was correct from the time of publication.

