# Supplementary material for: Smac mimetics synergize with immune checkpoint inhibitors to promote tumour immunity against glioblastoma
Source: Nat Commun. 2017 Feb 15;8:14278. doi: 10.1038/ncomms14278 (PMC5330852; doi:10.1038/ncomms14278)
Supplement: Supplementary Information — Supplementary Figures and Supplementary References [file ncomms14278-s1.pdf]

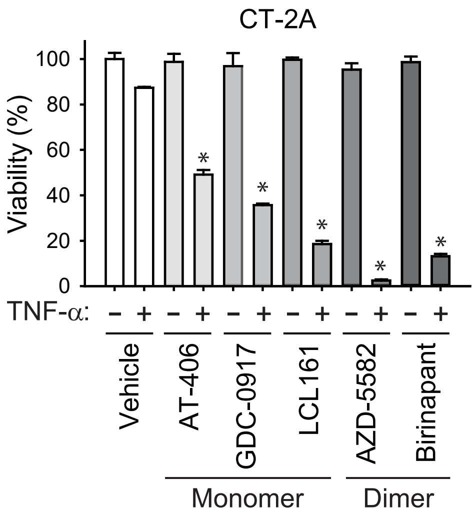

Supplementary Figure 1. SMCs potently synergize with TNF-α to induce the death of glioblastoma cells. Viability of mouse glioblastoma CT-2A cells to the treatment of 0.01% BSA or 0.1 ng mL^{-1} TNF-α and vehicle or 5 µM of the indicated monomeric or dimeric for 48 h. Viability was assessed by Alamar blue. Error bars, mean, s.d. n = 4. Representative data from two independent experiments using biological replicates. Statistical significance was compared to vehicle and BSA treatment using ANOVA using Dunnett's multiple comparison test. Significance is reported if $p < 0.0001$ (*).

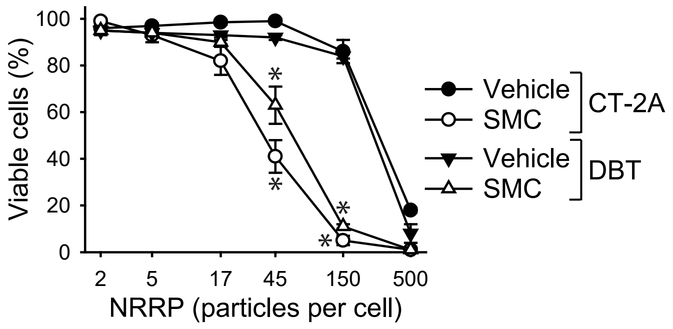

Supplementary Figure 2. SMCs synergize with non-replicating rhabdovirus-derived particles (NRRPs) to induce the death of glioblastoma cells.

Alamar blue viability assays of mouse glioblastoma CT-2A or DBT-cells to the treatment of vehicle or 5 µM LCL161 (SMC) and increasing numbers of NRRPs for 48 h. Error bars, mean, s.d. n = 3. Representative data from two independent experiments using biological replicates.

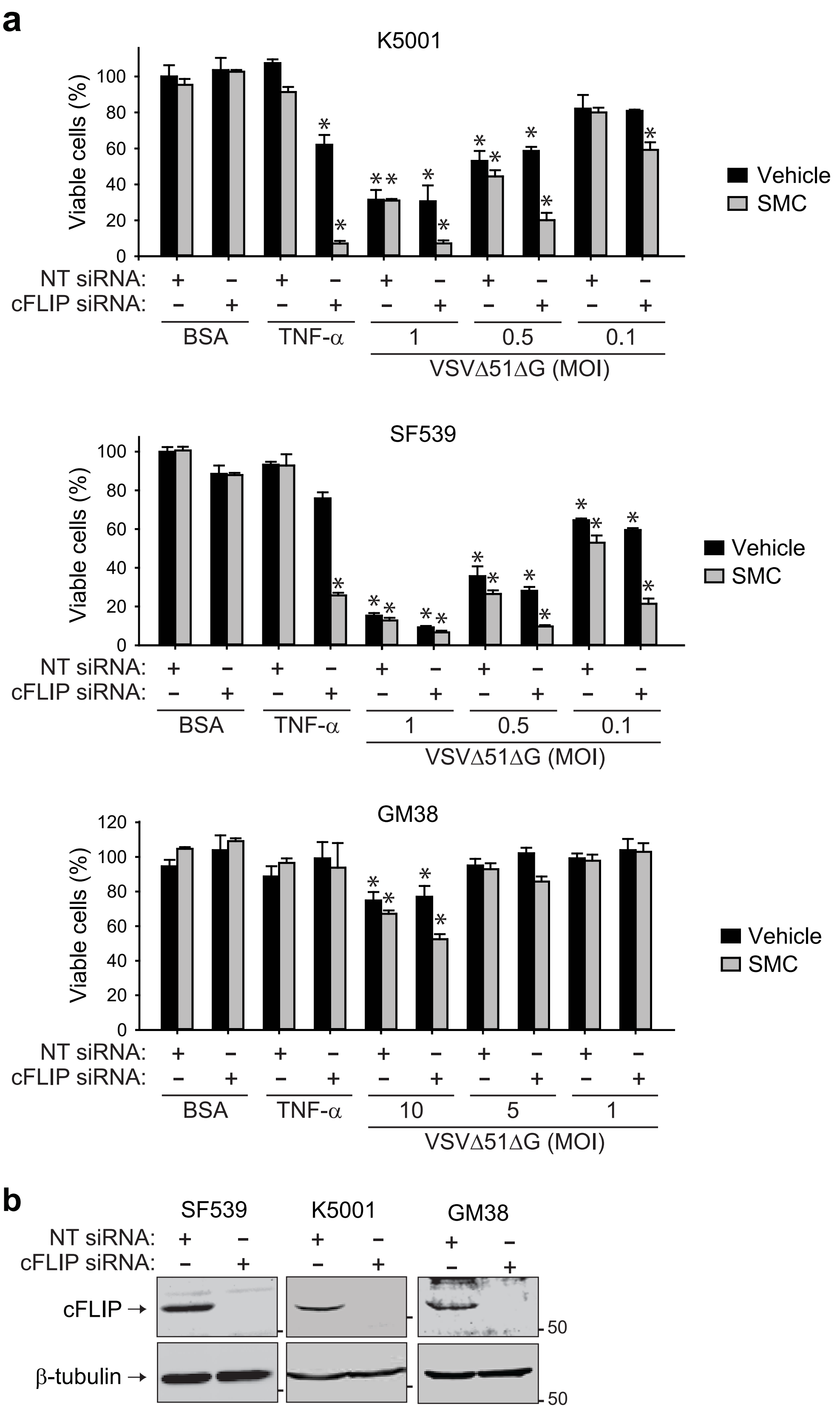

Supplementary Figure 3. Resistance to SMC-based combinations in glioblastoma cells is circumvented with downregulation of cFLIP.
(a) Primary mouse NF1-/+p53-/+ (K5001) or human (SF539) glioblastoma cells or human non-transformed cells (GM38) were transfected with nontargeting (NT) or cFLIP siRNA for 48 h and subsequently treated for 48 h with vehicle or 5 µM LCL161 (SMC) and BSA, 0.1 ng mL^{-1} TNF-α or the indicated MOI of a nonspreading version of VSVΔ51 (VSVΔ51ΔG). Viability was determined by Alamar blue. Error bars, mean, s.d. n = 4. Representative data from three independent experiments using biological replicates. Statistical significance was compared to vehicle and BSA treatment using ANOVA using Dunnett's multiple comparison test. Significance is reported if $p < 0.0001$ (*). (b) Efficacy of NT siRNA or siRNA targeting cFLIP from the experiment in (a).

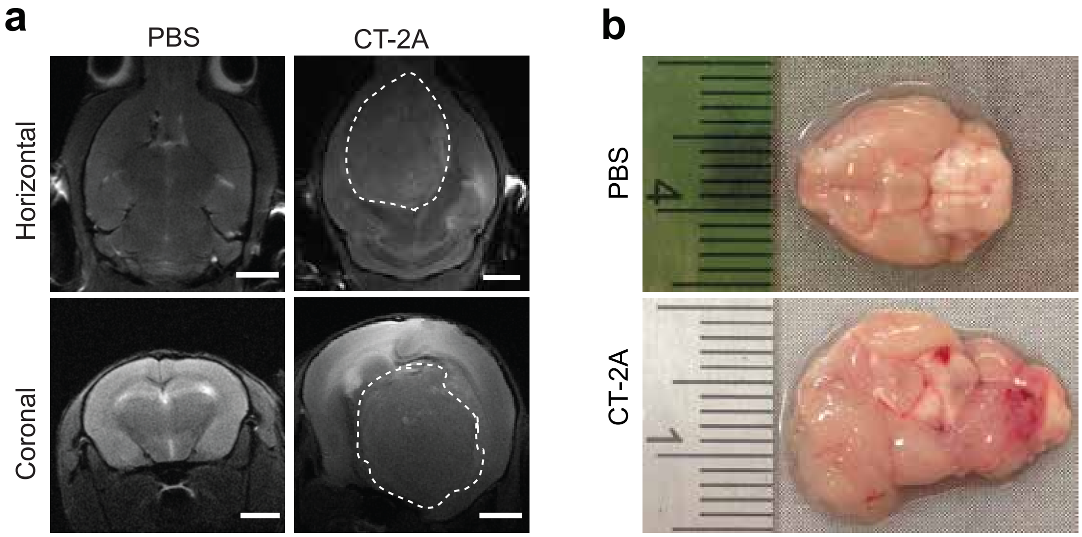

Supplementary Figure 4. Establishment of a mouse syngeneic orthotopic model of glioblastoma.
MRI (a) and gross (b) images of a C57BL/6 mouse injected intracranially with PBS or 5x104 CT-2A cells and sacrificed at 35 days post-implantation. Scale bar, 2 mm. Ruler is in cm with mm divisions.

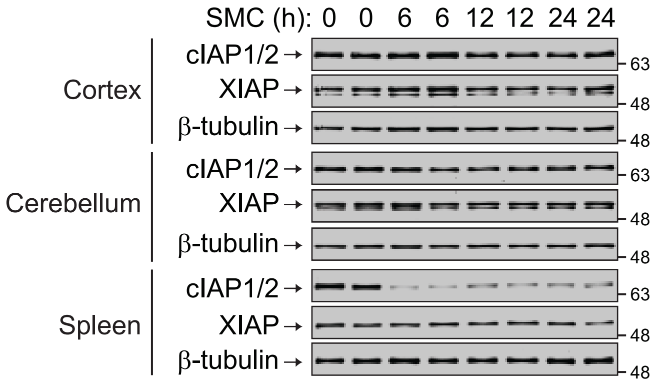

Supplementary Figure 5. SMC treatment does not induce the downregulation of the IAPs in brain tissue from non-tumor bearing mice.
Mice were treated with 75 mg kg^{-1} of LCL161 (SMC) for the indicated time, and tissues were processed for Western Blotting using the indicated antibodies. n = 2 for each timepoint.

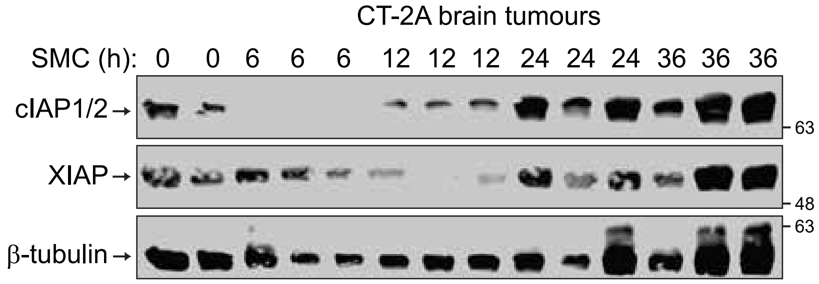

Supplementary Figure 6. Degradation of cIAP1/2 and XIAP in intracranial gliomas upon intratumoral injection of a SMC.
Mice with 21 d intracranial CT-2A tumors were treated by stereotactic injection (at the same coordinates as with the initial tumor implantation) with 10 µL of 100 µM LCL161 (SMC), and tumors were processed at the indicat-ed times post-treatment for Westerm blotting using the indicated antibodies. n = 2 for control and n = 3 for SMC-treated mice at the indicated post-treatment time.

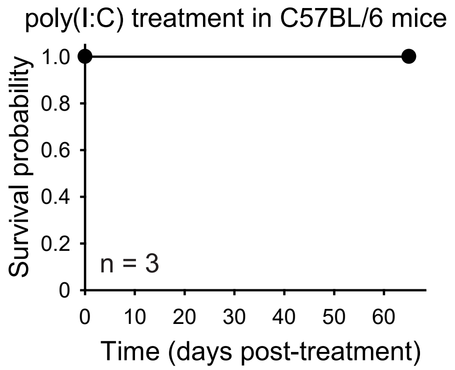

Supplementary Figure 7. Intracranial treatment of poly(I:C) does not cause mortality.
Kaplan-Meier curve depicting survival of mice that received three 10 μL intracranial treatments of 50 μg poly(I:C). Treatments were on days 0, 3, and 7.

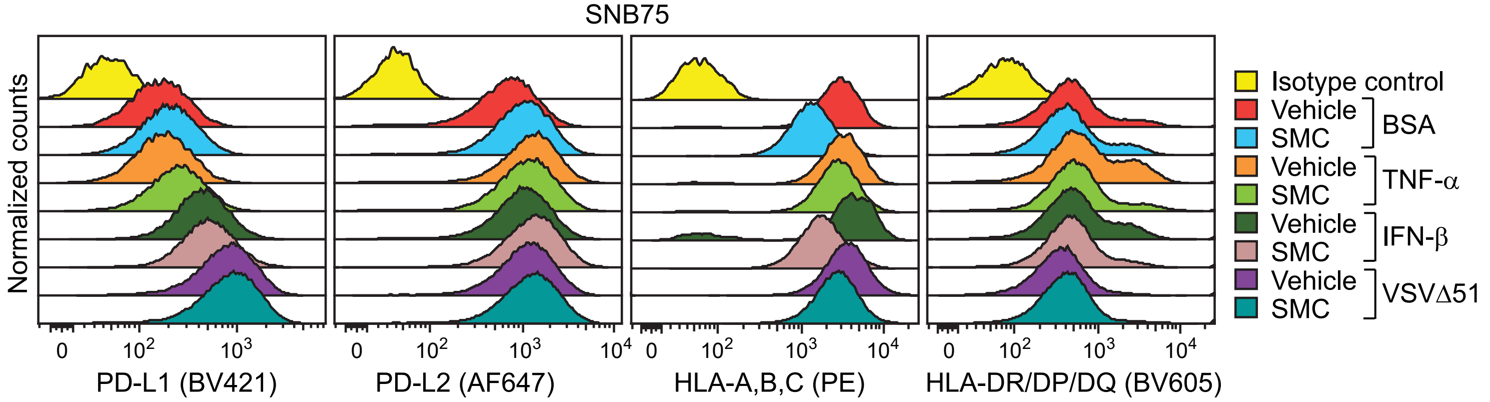

Supplementary Figure 8. SMC treatment does not abrogate expression of checkpoint inhibitor molecules or MHC I/II proteins.
SNB75 cells were treated for 24 h with vehicle or 5 μM LCL161 (SMC) and 1 ng mL^{-1} TNF-α, 250 U mL^{-1} IFN-β or 0.1 MOI of VSVΔ51, and viable cells (Zombie Green negative) were processed for flow cytometry using the indicated antibodies. Representative data from three independent experiments.

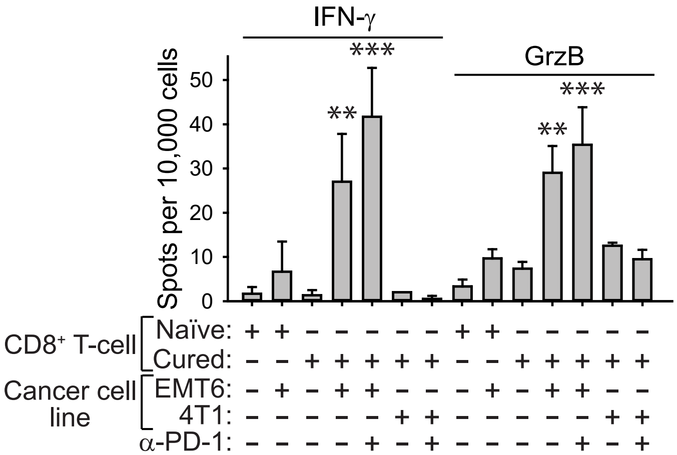

Supplementary Figure 9. CD8⁺ T-cells cells from mice previously cured of mammary tumors recognize the same tumor cell type.

CD8⁺ T-cells were enriched from naïve mice or mice previously cured of EMT6 tumors, and subjected to ELIS-pot assays for the detection of IFN-γ and GrzB. Cancer cells (EMT6, 4T1) were cocultured with CD8⁺ T-cells (12:1 ratio) and 10 µg mL^{-1} of control IgG or α-PD-1 for 48 h. Error bars, mean, s.d. n = 3 of mice for naïve or cured groups. Significance was compared to naïve CD8⁺ T-cell co-incubated with EMT6 cells as assessed by ANOVA with Dunnett's multiple comparison test. ** $p < 0.01$; ***, $p < 0.001$.

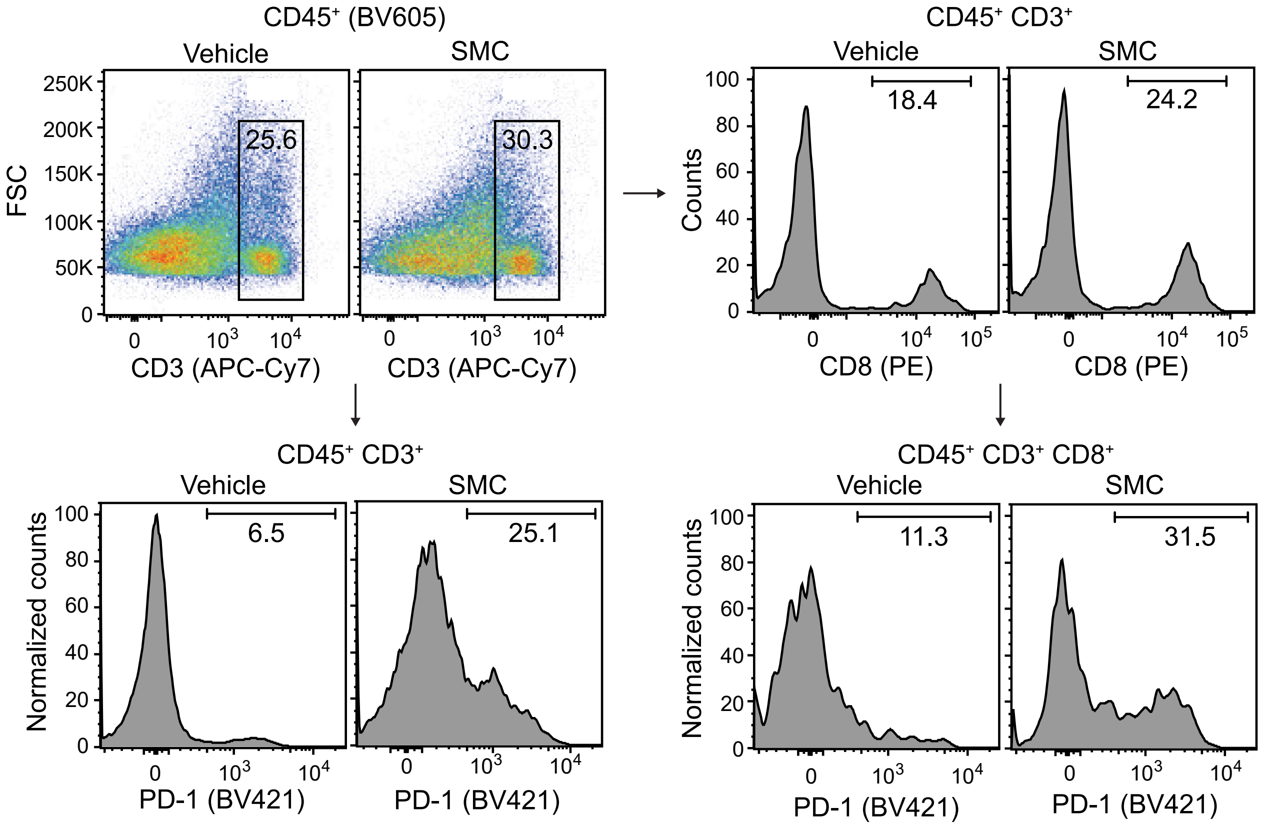

Supplementary Figure 10. SMC treatment leads to the upregulation of PD-1 in CD8 T-cells.

Mice bearing intracranial CT-2A tumors were treated with 75 mg kg^{-1} LCL161 orally (SMC) on post-implantation days 14, 16, 21 and 23. Viable cells from CT-2A tumors were processed for flow cytometry using the antibodies CD45 (BV605), CD3 (APC-Cy7), CD8 (PE) and PD-1 (BV421). Data represented as box and whisker plots are in Fig. 4b.

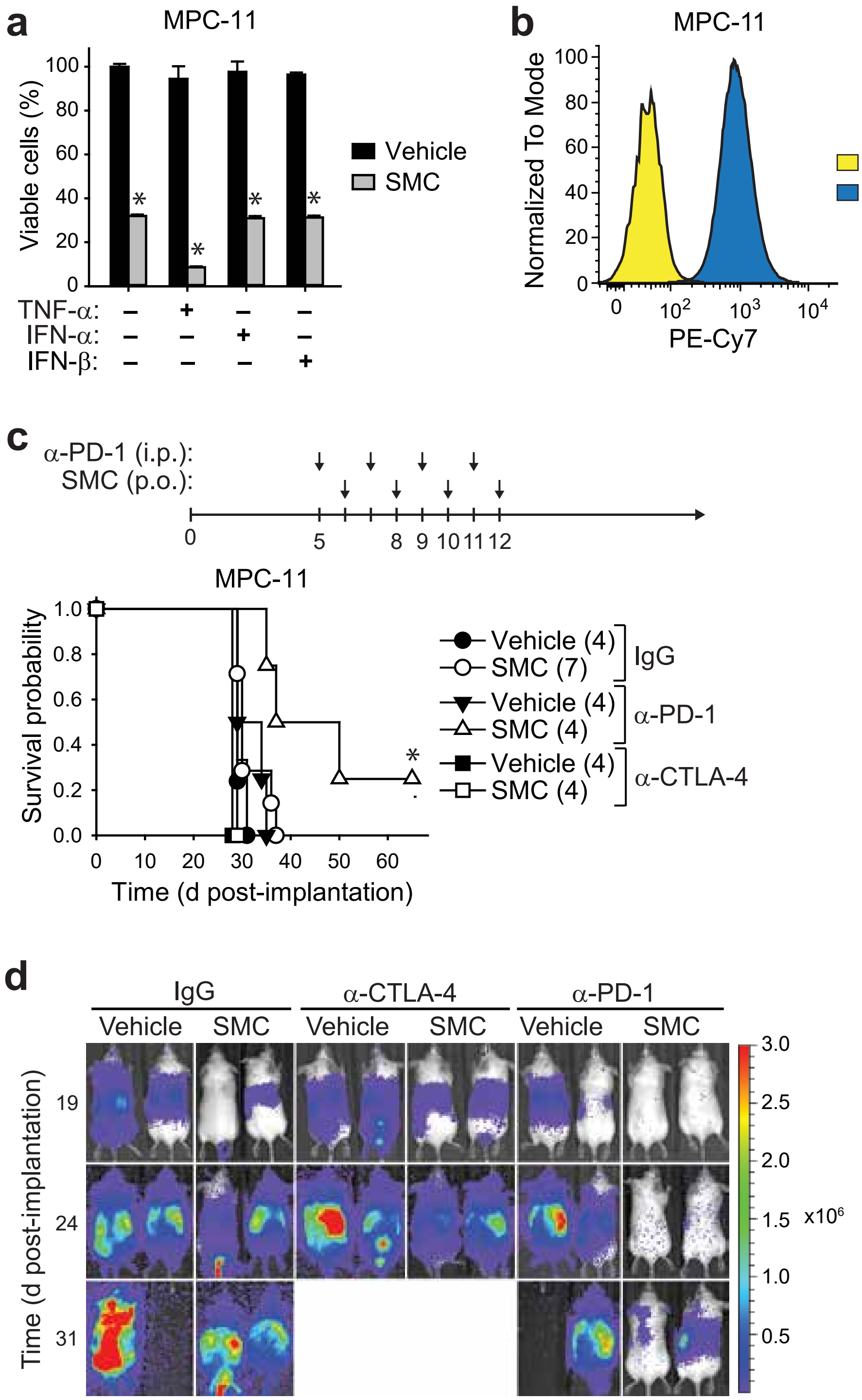

Supplementary Figure 11. SMCs synergize with immune checkpoint inhibitors for the treatment of a mouse model of multiple myeloma.

(a) MPC-11 cells were treated with vehicle or 5 μM LCL161 (SMC) and 0.1 ng mL^{-1} TNF-α, 250 U mL^{-1} IFN-α or 250 U mL^{-1} IFN-β. Viability was determined by Alamar blue at 48 h post-treatment. Error bars, mean, s.d. n = 4. Statistical significance was compared to vehicle and BSA treatment using ANOVA using Dunnett's multiple comparison test. Significance is reported if $p < 0.0001$ (***). Representative data from three independent experiments using biological replicates. (b) MPC-11 cells were dissociated and processed for flow cytometry with PE-Cy7-conjugated isotype IgG or PD-L1. Representative data from two independent experiments. (c) Mice were injected with murine MPC-11 cells intravenously, and 5 days later were treated at the indicated time points with combinations of vehicle or 75 mg kg^{-1} LCL161 orally and 250 μg of IgG, α-PD-1 or α-CTLA4 intraperitoneally. Data represents the Kaplan-Meier curve depicting mouse survival. Log-rank with Holm-Sidak multiple comparison: *, $p < 0.05$. Numbers in parentheses represent number of mice per group. Representative data from two independent experiments. (d) Representative IVIS images from mice described in (c) were acquired at indicated time points. Scale: p/sec/cm2/sr.

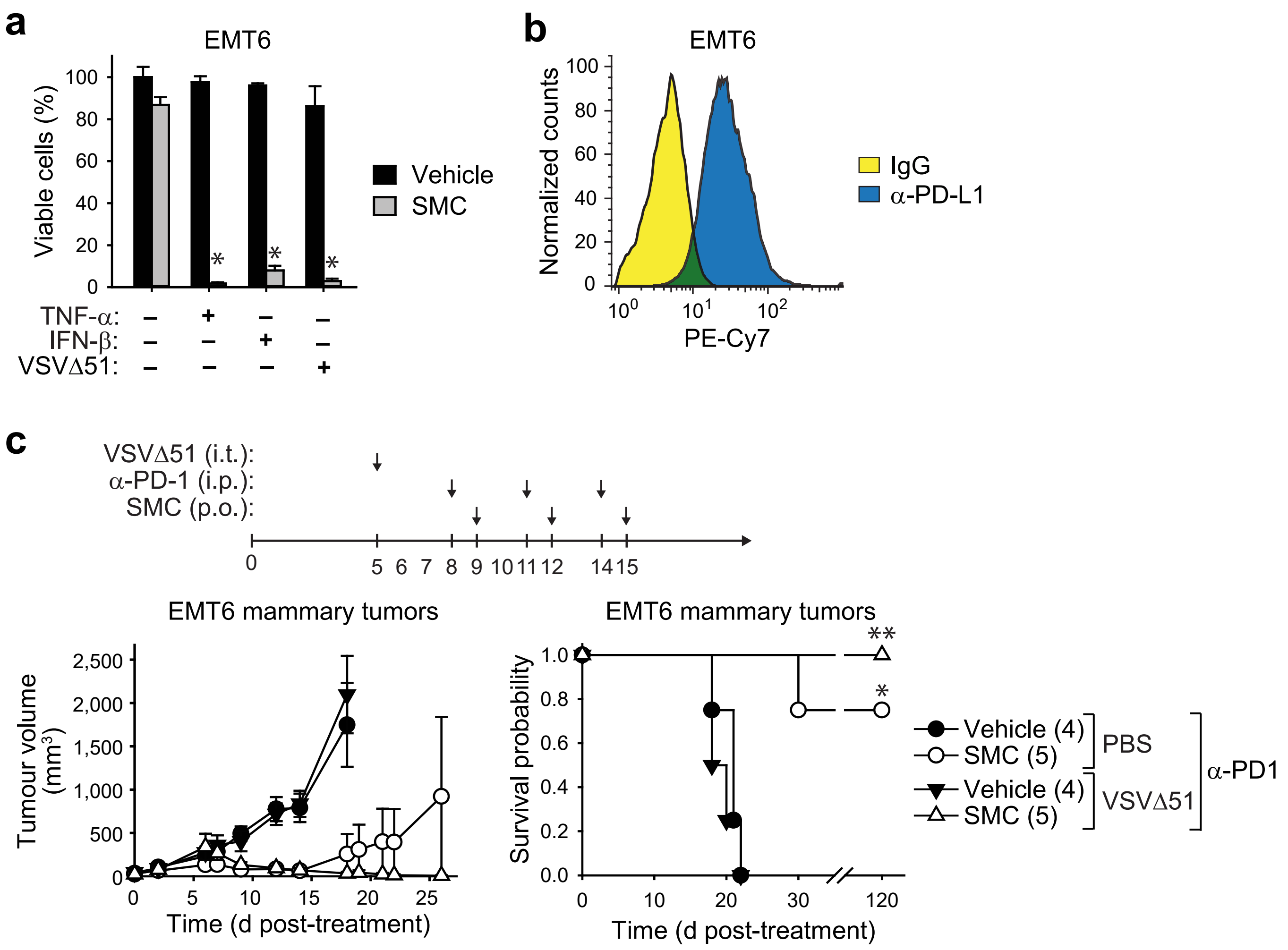

Supplementary Figure 12. The combination of SMCs with antibodies targeting immune checkpoint inhibitors in a mouse model of mammary cancer.

(a) Viability assay of EMT6 cells treated with vehicle or 5 μM LCL161 (SMC) and 0.1 ng mL^{-1} TNF-α, 250 U mL^{-1} IFN-β or 0.1 MOI of VSVΔ51 for 48 h. Error bars, mean, s.d. n = 4. Statistical significance was compared to vehicle and BSA treatment using ANOVA using Dunnett's multiple comparison test. Significance is reported if $p < 0.0001$ (***). Representative data from three independent experiments using biological replicates. (b) EMT6 cells were dissociated and processed for flow cytometry with PE-Cy7-conjugated isotype IgG or PD-L1. Representative data from three independent experiments. (c) Mice bearing ~100 mm^3 EMT6-Fluc tumors were treated at the indicated post-implantation times with PBS or $5x10^8$ PFU of VSVΔ51 intratumorally, and then with vehicle or 50 mg/kg LCL161 (SMC) orally and 250 μg of IgG or α-PD-1 intraperitoneally. Left panel depicts tumor growth. Error bars, mean, s.e,m. Right panel represents the Kaplan-Meier curve depicting mouse survival. Log-rank with Holm-Sidak multiple comparison: *, $p < 0.05$; **, $p < 0.01$. Numbers in parentheses represent number of mice per group.

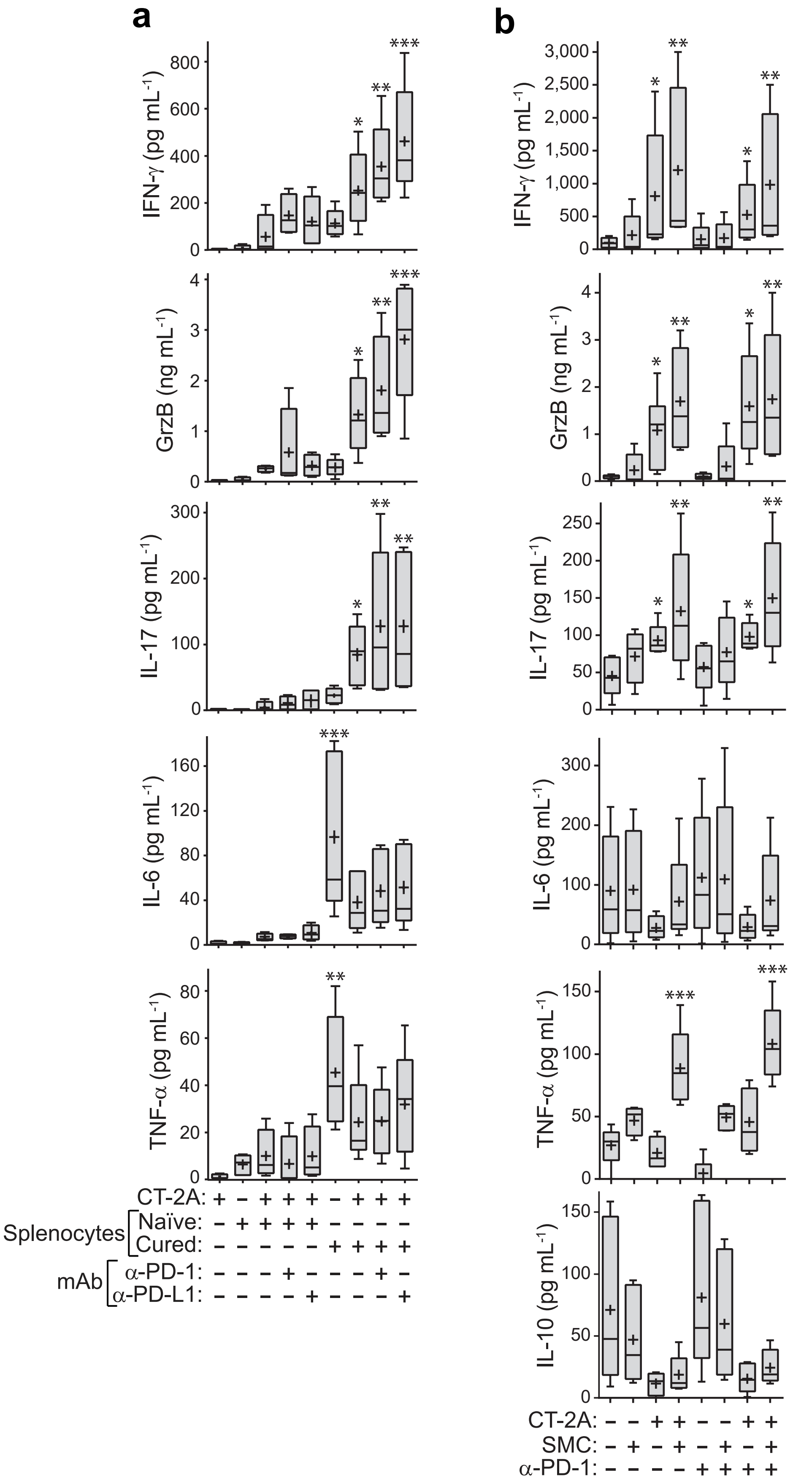

Supplementary Figure 13. The inclusion of SMCs increases the immune response in the presence of glioblastoma cells.

(a) The expression of the indicated factors was detected by ELISA from cell culture supernatants of CT-2A cells that were co-incubated for 48 h with splenocytes derived from naïve mice or mice previously cured with intracranial CT-2A tumors by SMC and anti-PD-1 cotreatment (1:20 ratio of CT-2A cells to splenocytes). Data represented as heat maps is in Fig. 5a. Crosses depicts mean, solid horizontal line depicts median, box depicts 25th to 75th percentile, and whiskers depicts min-max range of the values. Statistical significance was compared to naïve $CD8^+$ T-cell as assessed by ANOVA with Dunnett's multiple comparison test. *, $p < 0.05$; ** $p < 0.01$; ***, $p < 0.001$. (b) The indicated cytokines were determined by ELISA from CT-2A cells that were cocultured with splenocytes derived from naïve or cured mice and treated with vehicle or 5 µM LCL161 (SMC) for 48 h. Heat map plots are shown in Fig. 5a. . Crosses depicts mean, solid horizontal line depicts median, box depicts 25th to 75th percentile, and whiskers depicts min-max range of the values. Statistical significance was compared to vehicle and IgG treated T-cells as assessed by ANOVA with Dunnett's multiple comparison test. ** $p < 0.01$; ***, $p < 0.001$.

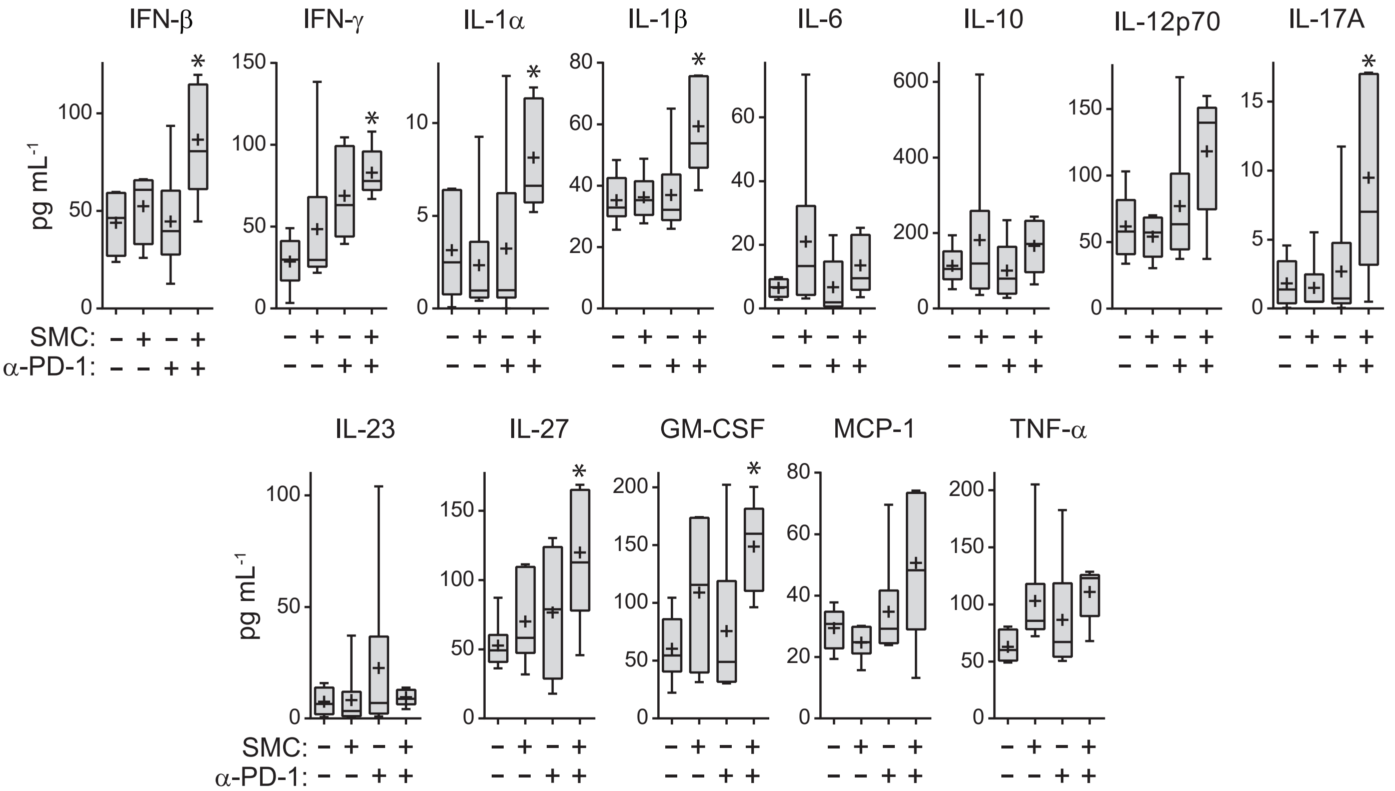

Supplementary Figure 14. Combinatorial SMC and immune checkpoint inhibitor treatment leads to the increased systemic presence of proinflammatory cytokines.

Serum from mice depicted in Fig. 6a was processed for multiplex ELISA for the quantitation of the indicated proteins. Crosses depicts mean, solid horizontal line depicts median, box depicts 25th to 75th percentile, and whiskers depicts min-max range of the values. Significance was compared to vehicle and IgG treated mice as assessed by ANOVA with Dunnett's multiple comparison test. *, $p < 0.05$. n = 6 for each treatment group. Data represented as heat maps is in Fig. 7e.

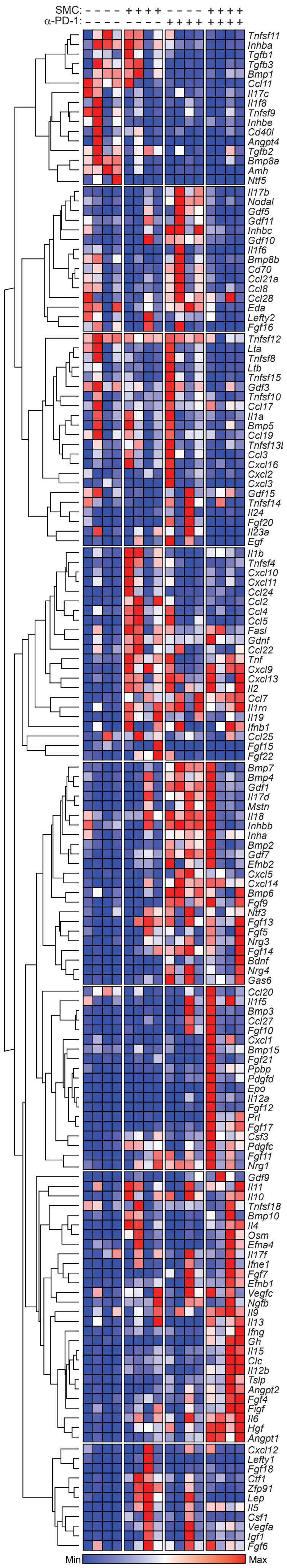

Supplementary Figure 15. Proinflammatory cytokine and chemoattractant chemokine gene signatures are upregulated with SMC and immune checkpoint inhibitor combinatorial treatment.

Mice were treated as in Fig. 6a, and intracranial CT-2A tumors were processed for quantitation of 176 cytokine and chemokine genes by RT-qPCR. Shown are normalized heat maps of major groups identified by hierarchical clustering. n = 4 for each treatment group.

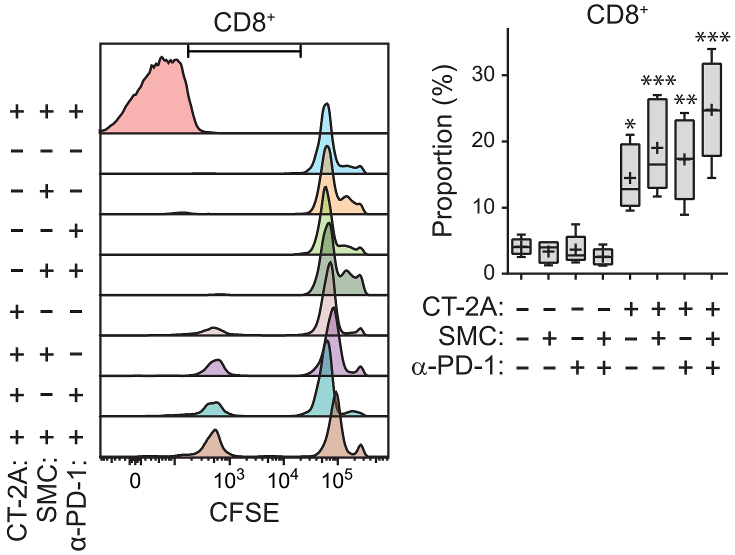

Supplementary Figure 16. SMCs enhance clonal expansion of $CD8^+$ T-cells in the presence of glioblastoma target cells.

Isolated splenic $CD8^+$ T-cells derived from mice previously cured of CT-2A tumors were loaded with CFSE and co-incubated with CT-2A cells (10:1 ratio) for 96 h in the presence of vehicle or 5 μM LCL161 (SMC) or 20 μg mL^{-1} of control IgG or anti-PD1. Viable cells were processed for flow cytometry. Significance was compared to vehicle and IgG treated mice as assessed by ANOVA with Dunnett's multiple comparison test. *, $p < 0.05$; **, $p < 0.01$; ***, $p < 0.001$. n = 5 for each treatment group.

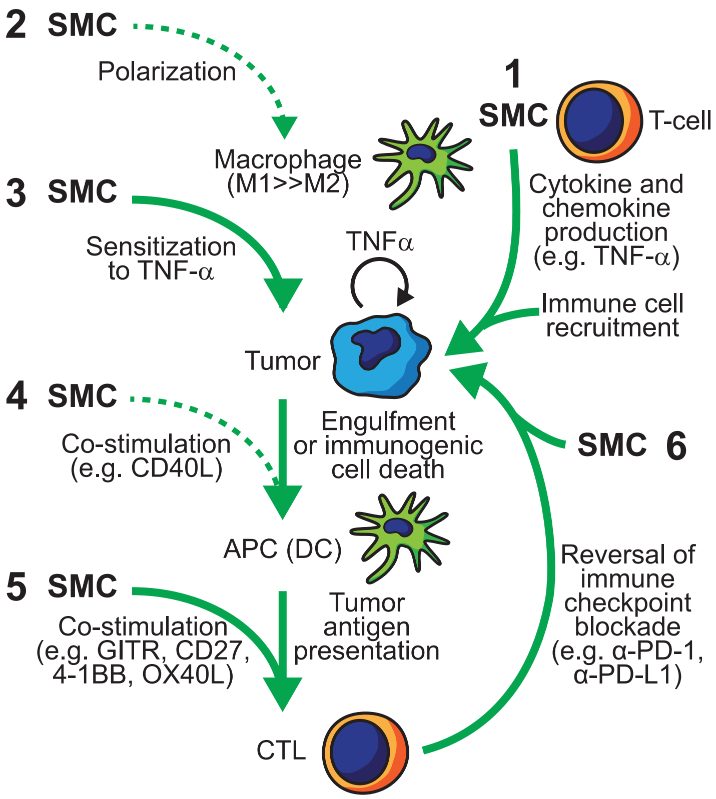

Supplementary Figure 17. SMCs are immunoregulatory drugs that act on tumor and immune cells to eradicate cancer through the innate and adaptive immune systems.
Shown is a model depicting the single agent and combinatorial immunomodulatory effects of Smac mimetics based on our results and previously published findings. The effects of IAP antagonism on these immune or tumor cells are outlined below: (1) SMCs stimulates the production of cytokines and chemokines from various immune cells, such as macrophages or T-cells, which results in infiltration of immune cells within the tumor microenvironment[1-3]. (2) SMC treatment decreases the immunosuppressive macrophage M2 population and concomitantly increases the pro-inflammatory M1 population[4]. (3) SMCs deplete cIAP1 and cIAP2 to sensitize tumors to death by immune ligands, such as TNF-α or TRAIL[1,5-8]. Tumor cell death is sensed by the immune system resulting in the priming of a cytotoxic T-cell (CTL) response. (4) SMCs stimulate the TNF/TNFR family member CD40L/CD40 signaling pathway on antigen-presenting cells (APCs) to promote the differentiation and maturation of dendritic cells (DCs) and macrophages[9]. APCs present tumor antigens to the immune system and further release cytotoxic inflammatory cytokines. (5) As a consequence of degrading cIAP1 and cIAP2 by SMC treatment, SMCs activate the alternative NF-κB pathway, removing the need for a TNF superfamily ligand (such as 4-1BB) and therefore providing a T-cell costimulatory signal[10, 11]. (6) SMCs have been shown to increase CTL and natural killer cell mediated cell death[12, 13]. Granzyme B-mediated cell death is blocked by the X-linked IAP, XIAP, and this block can be overcome by the mitochondrial release of Smac or by its drug mimic, SMC[13-15].

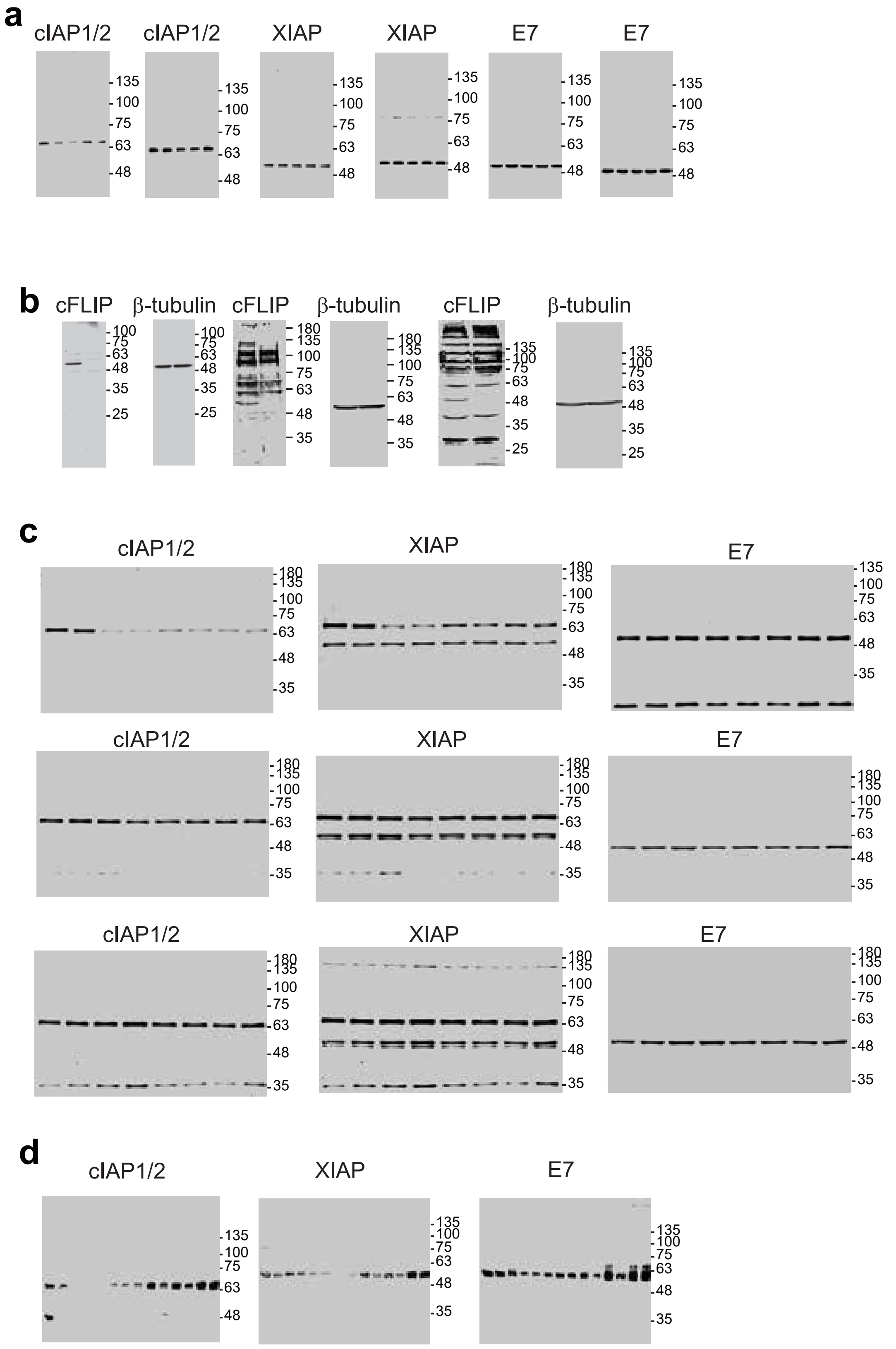

Supplementary Figure 18. Full-length blots pertaining to (a) Fig. 2a, (b) Supplementary Fig. 3b, (c) Supplementary Fig. 5 and (d) Supplementary Fig. 6.

Suppplementary References

1. Beug, S.T. et al. Smac mimetics and innate immune stimuli synergize to promote tumor death. *Nat Biotechnol* 32, 182-190 (2014).
2. Infante, J.R. et al. Phase I dose-escalation study of LCL161, an oral inhibitor of apoptosis proteins inhibitor, in patients with advanced solid tumors. *J Clin Oncol* 32, 3103-3110 (2014).
3. Erickson, R.I. et al. Toxicity profile of small-molecule IAP antagonist GDC-0152 is linked to TNF-alpha pharmacology. *Toxicological sciences : an official journal of the Society of Toxicology* 131, 247-258 (2013).
4. Lecis, D. et al. Smac mimetics induce inflammation and necrotic tumour cell death by modulating macrophage activity. *Cell death & disease* 4, e920 (2013).
5. Vince, J.E. et al. IAP antagonists target cIAP1 to induce TNFalpha-dependent apoptosis. *Cell* 131, 682-693 (2007).
6. Gaither, A. et al. A Smac mimetic rescue screen reveals roles for inhibitor of apoptosis proteins in tumor necrosis factor-alpha signaling. *Cancer Res* 67, 11493-11498 (2007).
7. Li, L. et al. A small molecule Smac mimic potentiates TRAIL- and TNFalpha-mediated cell death. *Science* 305, 1471-1474 (2004).
8. Fulda, S., Wick, W., Weller, M. & Debatin, K.M. Smac agonists sensitize for Apo2L/TRAIL- or anticancer drug-induced apoptosis and induce regression of malignant glioma in vivo. *Nat Med* 8, 808-815 (2002).
9. Muller-Sienerth, N. et al. SMAC mimetic BV6 induces cell death in monocytes and maturation of monocyte-derived dendritic cells. *PLoS One* 6, e21556 (2010).
10. Zarnegar, B.J. et al. Noncanonical NF-kappaB activation requires coordinated assembly of a regulatory complex of the adaptors cIAP1, cIAP2, TRAF2 and TRAF3 and the kinase NIK. *Nature Immunology* 9, 1371-1378 (2008).
11. Dougan, M. et al. IAP inhibitors enhance co-stimulation to promote tumor immunity. *The Journal of experimental medicine* 207, 2195-2206 (2010).
12. Kashkar, H. et al. XIAP targeting sensitizes Hodgkins Lymphoma cells for cytolytic T cell attack. *Blood* (2006).
13. Brinkmann, K. et al. Second mitochondria-derived activator of caspase (SMAC) mimetic potentiates tumor susceptibility toward natural killer cell-mediated killing. *Leukemia & lymphoma* 55, 645-651 (2014).
14. Goping, I.S. et al. Granzyme B-induced apoptosis requires both direct caspase activation and relief of caspase inhibition. *Immunity* 18, 355-365 (2003).
15. Ravi, R. et al. Resistance of cancers to immunologic cytotoxicity and adoptive immunotherapy via X-linked inhibitor of apoptosis protein expression and coexisting defects in mitochondrial death signaling. *Cancer Res* 66, 1730-1739 (2006).
